# Challenges and Barriers Affecting the Quality of Triage in Emergency Departments: A Qualitative Study

**DOI:** 10.31661/gmj.v8i0.1619

**Published:** 2019-10-12

**Authors:** Mostafa Bijani, Ali Asghar Khaleghi

**Affiliations:** ^1^Department of Medical Surgical Nursing, Fasa University of Medical Sciences, Fasa, Iran; ^2^Non Communicable Diseases Research Center (NCDC), Fasa University of Medical Sciences, Fasa, Iran

**Keywords:** Triage, Nurses, Qualitative Research, Emergency Department

## Abstract

**Background:**

Correct and fast triage is the key to successful performance in emergency departments. Various factors can affect the quality of triage; therefore, the present study was conducted to identify and explore the existing challenges in triage units in emergency departments.

**Materials and Methods:**

The present study was a qualitative exploratory work conducted via the content analysis approach. Data were collected using in-depth, semi-structured interviews, and focus group interviews. Accordingly, 22 in-depth, semi-structured interviews were conducted in with 18 triage nurses and four emergency medicine specialists on a face-to-face basis.

**Results:**

The challenges related to triage nurses fall into two subcategories; lack of clinical competency and psychological capabilities. The challenges related to emergency management consist of challenges in human resources management, structural, and performance.

**Conclusion:**

The challenges existing in triage units are influenced by factors related to triage nurses and emergency management. Emergency administrators can improve the effectiveness and quality of triaging patients by empowering triage nurses and removing structural problems in triage units.

## Introduction

Nowadays, in healthcare systems, triage is considered as an inseparable part of emergency management in hospitals and also as an index in assessing and validating emergency services in hospitals [1]. Triage is defined as prioritizing patients according to the seriousness of their conditions and providing the most appropriate clinical care to the largest possible number of patients in the shortest possible time [2]. If a patient is not triaged properly in the first few minutes of entering the hospital and does not receive effective clinical care, the highly advanced specialist wards may not be able to provide effective care to the patient in the following days [3]. In the studies conducted in other parts of the world, it has been established that the long waiting periods and patients’ stay in the emergency ward are the results of inefficient workflow in the emergency department witch is, in turn, due to lack of an effective triage unit to prioritize patients [4]. Correct and fast triage is the key to successful performance in an emergency ward, If not equipped with the necessary skills triage nurse can makes mistake in prioritizing patients [5]. Correct triage will increase the quality of patients’ care services, reduces the waiting time and patients’ length of stay, lessen mortality and ultimately lower treatment costs [6]. O’Connor *et al*. (2014) state that failure to perform triage correctly and effectively, makes the emergency ward crowded, leads to delays in the transport of patients from the emergency ward to other hospital wards and results in the dissatisfaction of patients and patients’ companions [7]. Studies conducted on triage in the emergency departments of hospitals in Iran indicated that the quality of triage is not satisfactory, and nurses’ knowledge and skills in this area are inadequate [8-9]. There is the need for plans to the weaknesses and strengths and improve the quality of triage in the emergency wards of hospitals through studying and determining the challenges and problems triage nurses are faced with them. There have been several quantitative studies on triage in the emergency departments of hospitals in Iran; yet, the value of knowledge in nursing depends on its relationship with understanding human perspectives and experiences. Achieving such an understanding requires research methods that provide the possibility to explore and discover all these experiences comprehensively, thus the need for qualitative studies. In view of a lack of a qualitative work of research in this area, the present study was conducted to investigate the challenges and barriers affecting the quality of triage in an educational hospital affiliated to Fasa University of Medical Sciences in the southeast of Iran.

## Materials and Methods

The present work of research was a qualitative study conducted using the conventional content analysis approach. In this method, which is used for a subjective interpretation of the content of textual data, the contents of texts are analyzed for extracting main themes and patterns from the data [10]. Content analysis is more than a mere extraction of obvious content from textual data; it enables the researcher to identify key concepts and hidden patterns in the content of the data provided by the participants in a study [11].

### Sample and Setting

In the present study, 18 (10 males and 8 female) nurses, and 4 (2 males and 2 female) emergency medicine specialists were selected through the purposeful sampling method from the triage unit of an educational hospital, affiliated to Fasa University of Medical Sciences in the southeast of Iran from February 2019 to May 2019. The inclusion criteria for the nurses were having at least a bachelor’s degree in nursing, having at least one year’s experience of work in the triage ward, and willing to participate in the study. The inclusion criteria for emergency medicine specialists were having at least one year’s work experience in the emergency ward and willing to participate in the study.

### Data Collection

Data were collected using individual and focus group interviews; for this purpose, 22 semi-structured, face–to face, in-depth interviews were performed with 18 triage nurses and four emergency medicine specialists. Each participant was interviewed in one or two sessions each lasting from 45 to 60 minutes. In addition, two focus group meetings were held with 7 triage nurses—the meetings lasted from 90 to 120 minutes. The interviews were continued until in-depth data were obtained, and the process of participant selection was continued to the point of data saturation.After the necessary arrangements had been made with the head of the department and the participants’ willingness had been established, the interviews were conducted in the rec-room or the conference room of the hospital. At the start of each interview, after stating the purpose of the study, the interviewer asked the subject a few descriptive questions about his/her work experience, academic degree, and type of employment; afterward, structural questions were asked such as: “Can you describe your typical workday in the triage ward? “. “What challenges and problems are you facing in the triage ward?” and “What factors affect the quality of triage? “. Also, follow-up questions were used to increase the clarity of the information provided by the participants, for example: “Can you elaborate on what you said?” “What do you mean by this?” “Can you give an example or talk about your experience?” Manuscripts of the interviews were typed immediately after completion, and each interview manuscript was read several times. The researchers, while being present in the field to interview the participants, also recorded their observations of the place, the interactions of the participants with their colleagues and their conversations, and their nonverbal behaviors. The field notes were used as a complement to collected data from the interviews and to fill the gaps in the categories and concepts derived from the data. Therefore, to prevent misinterpretations of and to add to the richness of the data, the researchers field notes immediately after each interview. The filed notes were analyzed concurrently with the data from the interviews. Finally, the data were analyzed using MAXQDA software (version 10, VERBI, Germany).

### Ethical Considerations

The research plan for the present study was approved by the Ethics Committee and Research Council of Fasa University of Medical Sciences (IR.FUMS.REC.1397.180). Before being interviewed, the participants were informed about the purpose of the study, the voluntary nature of their participation, the data collection methods and the reasons for recording the data, the roles of the researchers and participants, the confidentiality of data and anonymity of the participants. They were then asked to complete the informed consent form if they were willing to participate in the study. The Participants were informed that they had the right to withdraw from the study at any time.

### Data Analysis

The conventional content analysis method as suggested by Graneheim and Lundman was used for analysis [12]. The five steps of the content analysis method are as follows:

1- Writing manuscript of the entire interview immediately after each interview,

2- Reading the entire interview manuscripts to achieve a general understanding,

3- Determining units of meaning and initial codes,

4- Classifying similar initial codes in more comprehensive categories based on similarities and differences,

5- Choosing a proper title which would represent each obtained category [12].

### Rigor

The proposed criteria proposed by Guba and Lincoln (1985) were used to ensure the trustworthiness of the collected data [13]. To confirm the credibility of the obtained data, the researchers employed the methods of prolonged engagement and member check. For this purpose, a summary of the manuscript of each interview was given to the participants to confirm the validity of the researcher’s perceptions. The ability of the data was assessed via the peer check method. For this purpose, all the coded data and categories were reviewed and examined by four nursing professors who were experts in the field of qualitative research. In the present study, to achieve dependability of data, the researchers employed a combination of the methods of data collection (semi-structured interviews, focus group interviews and field notes) and the audit trail (observance of the correct technique for interviewing, accuracy in the note-taking and analysis by peers). Also, sampling was done with maximum variance. For the purpose of ensuring the transferability of data, complete and accurate descriptions of the research method, the participants’ characteristics, the data collection, data analysis method, along with examples of the participants’ statements were provided, to make the tracking of the trail of research possible [14].

## Results

The mean age of the nurses was 31.60 ± 4.27 years, and the mean of their work experience was 8.10 ± 3.93 years. The mean age of the emergency medicine specialists was 40.50 ± 3.53 years, and the mean of their work experience was 7.50 ± 2.12 years. The analysis of the data obtained from interviewing the participants yielded 700 codes, two categories, and five subcategories (Table-1).

### 1. Challenges Related to Nursing Personnel

#### 1.1. Lack of Clinical Competency

From the participants’ viewpoint, a triage nurse should have professional capabilities, including adequate knowledge about how to triage patients. Other capabilities referred to by the participants were clinical experience, sufficient clinical knowledge in the field of physiopathology of diseases and high-risk emergencies, clinical skill to carry out correct and fast clinical measures in high-risk emergency conditions, the ability to check patient history and do physical examinations, critical thinking skills, clinical decision-making skills, clinical intuition and inter-professional communication skills. One of the participants stated, *‘Some of our colleagues do not have enough clinical knowledge and experience in triage, and make mistakes in prioritizing which makes the triage ward overcrowded and the patients and their companions dissatisfied” (Nurse 4, female).* Regarding clinical knowledge, one participant mentioned, *“A triage nurse should have complete knowledge of diseases; for example, he/she should know what the symptoms CVA are? How it is treated? When I, as a triage nurse, do not have clinical knowledge about diseases, how can I evaluate patients well and perform a correct triage?” (Nurse 9, male).* One emergency medicine specialists stated, *“Having clinical knowledge is a very essential capability for a triage nurse. If a triage nurse does not have clinical knowledge, he/she cannot identify the patient’s priorities well. For example, in dealing with multiple trauma patients, if the nurse does not know high - risk emergencies well, he/she cannot determine high-risk priorities in such a patient”.* About the clinical skill, one of the participants stated, *“From my point of view, a triage nurse, in addition to having clinical knowledge, should be able to act quickly and possess the clinical skill, be able to evaluate vital signs quickly and accurately and perform an accurate assessment. There have been cases where patients were triaged wrongly because the triage nurse did not accurately assess the vital signs “ (Nurse 14, female).*

#### 1.2. Lack of Psychological Capability

From the participants’ viewpoint, having emotional stability and high tolerance are among the most important psychological capabilities in a triage nurse. Psychological capability is defined as a triage nurse’s ability to adapt him/herself to the severe conditions of the emergency ward have a high tolerance, calm and maintain mental concentration in critical conditions, and control his/her emotional behaviors. One of the emergency medicine specialists stated, *“Having a high tolerance capacity is very essential for a triage nurse. I have witnessed cases in which triage nurses with good knowledge and skill, lost control and failed to manage matters in an emergency in critical conditions and when the emergency room became very crowded” (Nurse 12, male).* Another participant said, *“Some colleagues do not have emotional stability and get angry too easily and argue with patients and their companions. This has a negative impact on patients’ satisfaction and the quality of triage” (Nurse, 7 female).*

### 2. Management Challenges

#### 2.1. Challenges Related to Human Resources

One of the important management challenges mentioned by the participants was the shortage of personnel. From the participants’ viewpoint, the triage unit in a hospital is a highly stressful environment with high workload, fatigue and work overload can lead to nurses loss of concentration and mistakes in prioritizing patients, which, in turn, leads to overcrowding in triage units and the dissatisfaction of patients and their companions.

#### 2.2. Structural Challenges

According to the participants’ viewpoint, the absence of appropriate and adequate physical space, and also the shortage of personnel and the inefficiency of the security section were the most important structural challenges in the triage ward. One of the participants mentioned, *“When several patients visit the triage unit simultaneously, because we don’t have enough space here, we are not able to provide services to all of them, and some patients may get missed”* (*Nurse15, male).* Another participant stated, *“Because of the shortage of personnel and the poor performance of the security staff, a large number of patients’ companions enter the triage unit, causing overcrowding and disruption in the nurses’ practice*” *(Nurse 10, female).*

#### 2.3. Performance Challenges

From the participants’ viewpoint, lack of motivation the administrators’ failure to encourage the personnel, absence of specific instructions and policies for triaging patients, as well as lack of holding training and specialized workshops to empower triage nurses were major performance challenges.One of the participants mentioned, *“The system of encouragement and rewards is very weak. There is no difference between someone who works well and someone who does not. If you work well, they’ll say you are doing your job, and if you make a mistake, they will punish you”* (*Nurse 13, male).* Regarding the absence of instructions for triage, one of the participants said,* “There is no single clear manual for triage at all. We are faced with double standards: If we triage all patients, the emergency doctors will protest that the emergency room has gotten too crowded and we do not have a vacant bed. If we do not triage all patients, the patients will complain, and the hospital management will punish and reprimand us”* (*Nurse 8, female).* One of the participants said about empowering triage nurses, *“Holding workshops and specialized training courses by managers is essential for promoting the professional knowledge triage nurses and keeping them up-to-date scientifically”* (*Nurse11, male).*

## Discussion

The present qualitative study was conducted to understand the experiences of triage nurses about the challenges and barriers affecting the quality of triage. Insufficient professional competency was referred to as one of the important challenges in triage from the participants’ viewpoint. The Emergency Nursing Association emphasize the emergency ward of a hospital is an unpredictable environment and a large number of patients with various problems visit, it is essential that emergency nurses have the necessary professional capabilities [15]. Duko *et al*. (2019) state that having knowledge about triage protocols and adequate clinical knowledge in the field of identification of diseases and emergency conditions is the key to prioritizing patients accurately and performing effective and quality triage [16]. The results of studies conducted in Iran show that triage nurses’ professional knowledge is unsatisfactory and since triage nurses do not have sufficient knowledge with regard to prioritizing patients and identifying diseases, they make mistakes in their practice witch, in turn, leads to patients’ satisfaction and overcrowding in triage units in the emergency wards of hospitals [17]. A study by Aloyce *et al*. (2014) in Tanzanian hospitals show that most triage nurses, due to lack of knowledge about triage, make mistakes in prioritizing patients and place patients on a lower or higher level than their actual status. According to the results of their study, triage nurses placed 25% of patients on a lower level, and 42% of patients on a higher level than their actual levels [18]. Likewise, the study results of Rahmati *et al* (2013) showed that due insufficient speed and skill in assessment of patient’s status, triage nurses cause overcrowding and patient dissatisfaction in the emergency ward [19]. Lack of psychological capability was another major challenge referred to by the participants. According to the experiences of the participants, a triage nurse should have high resilience to adapt him/herself to severe and stressful conditions. Considering that the patient and his/her companions are in the worst emotional state when referred to emergency, their first encounter with triage nurses may be characterized by abnormal and aggressive behaviors; therefore, triage nurses should have psychological capabilities, including tolerance and emotional stability so that they can control their feelings and emotions and treat patients with patience and tolerance. The study of Lin *et al*. (2019) shows that emergency nurses should have the resilience to effectively use their capabilities in critical and emergency conditions and take effective clinical measures [20]. The triage unit in the emergency ward of a hospital is a very stressful work environment, and triage nurses, in addition to enduring the everyday physical and psychological stresses that exist in all hospital wards, are exposed to additional challenges, such as unpredictability in the number of patients at any time, and rapid and critical changes in patients’ conditions; therefore, the psychological empowerment of triage staff against occupational stresses has an important role in upgrading the quality of their professional life and, subsequently the quality of care provided by them [21]. Personnel shortage was another major administrative challenges from the participants’ viewpoint. Rosenberg *et al*. (2019) state that shortage of nursing personnel is a global problem, which both developed and developing countries are dealing with it. Lack of nursing personnel, in addition to its adverse effects on the quality of care, other problems, including quitting jobs and occupational burnout among nurses [22]. Similarly, Li *et al*. (2018) indicated that shortage of nursing staff and increase in workload and the resultant fatigue in an emergency ward cause patient dissatisfaction and lower the quality of health care services [23].

## Limitations

Given the nature of qualitative studies, the transferability of the results of the present study was limited to the environment of a triage unit specifically; therefore, more studies are recommended to identify the challenges and barriers affecting the triage quality in emergency departments in other countries.

## Conclusion

Based on the findings of the present study, the challenges in a triage unit in the emergency ward of a hospital consist of factors related to the nursing personnel and factors associated with the management of the emergency ward. Emergency administrators can use the results of the study to eliminate the challenges existing in hospitals triage units; thereby, improving the effectiveness and quality of triage and patient satisfaction.

## Acknowledgment

This present article was extracted from a research project approved by Fasa University of Medical Sciences (grant number: 97220). The authors are indebted to Fasa University of Medical Sciences for their financial support of this research.

## Conflict of Interest

The authors declare that they have no conflicts of interest.

**Table 1:** The Extracted Categories and Subcategories

**Categories**	**Subcategories**
Factors related to triage nurses	Lack of clinical competency
	Lack of psychological capability
Factors related to the management of emergency ward	Challenges in human resources management
	Structural challenges
	Performance challenges

## References

[1] Yarmohammadian MH, Rezaei F, Haghshenas A, Tavakoli N (2017). Overcrowding in emergency departments: A review of strategies to decrease future challenges. J Res Med Sci.

[2] Love RA, Murphy JA, Lietz TE, Jordan KS (2012). The effectiveness of a provider in triage in the emergency department: a quality improvement initiative to improve patient flow. Adv Emerg Nurs J.

[3] Jordi K, Grossmann F, Gaddis GM, Cignacco E, Denhaerynck K, Schwendimann R (2015). Nurses’ accuracy and self-perceived ability using the Emergency Severity Index triage tool: a cross-sectional study in four Swiss hospitals. Scand J Trauma Resusc Emerg Med.

[4] Robinson DJ (2013). An integrative review: Triage protocols and the effect on ED length of stay. J Emerg Nurs.

[5] Buschhorn HM, Strout TD, Sholl JM, Baumann MR (2013 Sep). Emergency medical services triage using the emergency severity index: is it reliable and valid?. J Emerg Nurs.

[6] Ganley L, Gloster AS (2011). An overview of triage in the emergency department. Nursing Standard.

[7] O’Connor E, Gatien M, Weir C, Calder L (2014). Evaluat-ing the effect of emergency department crowdingon triage destination. Int J Emerg Med.

[8] Fathoni M, Sangchan H, Songwathana P (2010). Triage knowledge and skills among emergency nurses in East Java Province, Indonesia. Aust Emerg Nurs J.

[9] Rahmati H, Azmoon M, Meibodi MK, Zare N (2013). Effects of Triage Education on Knowledge, Practice and Qualitative Index of Emergency Room Staff: A Quasi- Interventional Study. Bull Emerg Trauma.

[10] Bengtsson M. How to plan and perform a qualitative study using content analysis. Nursing Plus Open. 2016 ; 2: 8-14.

[11] Vaismoradi M, Turunen H, Bondas T (2013). Content analysis and thematic analysis: Implications for conducting a qualitative descriptive study. Nursing and Health Sciences.

[12] Graneheim UH, Lundman B (2004). Qualitative content analysis in nursing research: concepts, procedures and measures to achieve trustworthiness. Nurse Education Today.

[13] Loh J (2013). Inquiry into Issues of Trustworthiness and Quality in Narrative Studies: A Perspective. The Qualitative Report.

[14] Cypress BS (2017). Rigor or Reliability and Validity in Qualitative Research: Perspectives, Strategies, Reconceptualization, and Recommendations. Dimens Crit Care Nurs.

[15] Ghanbari A, Hasandoost F, Lyili EK, Khomeiran RT, Momeni M (2017). Assessing Emergency Nurses’ Clinical Competency: An Exploratory Factor Analysis Study. Iran J Nurs Midwifery Res.

[16] Duko B, Geja E, Oltaye Z, Belayneh F, Kedir A, Gebire M (2019 14). Triage knowledge and skills among nurses in emergency units of Specialized Hospital in Hawassa, Ethiopia: cross sectional study. BMC Res Notes.

[17] Reisi Z, Saberipour B, Adienh M, Hemmatipour A, Abdolahi Shahvali E (2018). The level of awareness of the emergency department nurses of the triage principles in teaching hospitals. J Nurs Midwifery Sci.

[18] Aloyce R, Leshabari S, Brysiewicz P (2014). Assessment of knowledge and skills of triage amongst nurses working in the emergency centres in Dar es Salaam, Tanzania. African Journal of Emergency Medicine.

[19] Rahmani F, Sepehri Majd P, Ebrahimi Bakhtavar H, Rahmani F (2018). Evaluating the accuracy of emergency nurses in correct triage using emergency severity index triage in Sina hospital of Tabriz: a cross-sectional analysis. J Emerg Prac Trauma.

[20] Lin CC, Liang HF, Han CY, Chen LC, Hsieh CL. Professional resilience among nurses working in an overcrowded emergency department in Taiwan. Int Emerg Nurs. 2019 Jan;42:44-50. 10.1016/j.ienj.2018.05.005 29954706

[21] Mohseni M, Seyedin H, Hosseini AF (2019). Relationship between Psychological Empowerment of Managers and Performance Accountability in Hospitals Affiliated to Tehran University of Medical Sciences, Tehran, Iran. Journal of Patient Safety and Quality Improvement.

[22] Rosenberg K (2019). RN Shortages Negatively Impact Patient Safety. Am J Nurs.

[23] Li H, Cheng B, Zhu XP. Quantification of burnout in emergency nurses: A systematic review and meta-analysis. Int Emerg Nurs. 2018 ;39:46-54. 10.1016/j.ienj.2017.12.005 29361420

